# Supplementary material for: Effect of physiotherapy interventions on pain management, function and quality of life in patellofemoral pain syndrome: A systematic review protocol
Source: PLoS One. 2024 Dec 19;19(12):e0312279. doi: 10.1371/journal.pone.0312279 (PMC11658526; doi:10.1371/journal.pone.0312279)
Supplement: Attachment — Submitted filename: Rebuttal Letter PLOS ONE.docx [file pone.0312279.s003.docx]

**Rebuttal Letter**

**Title:** Effect of Physiotherapy Interventions on Pain Management, Functional Recovery and Quality of Life in Patellofemoral Pain Syndrome: A Systematic Review Protocol

**Manuscript ID:** PONE-D-24- 27461

Dear Editors,

Thank you for your feedback regarding our manuscript. We understand that the manuscript did not meet the criteria for a Registered Report Protocol. We have revised our manuscript accordingly and are now submitting it as a study protocol as recommended by the editor.

Response to Editorial Comments

Editor Comment: The submission does not meet the criteria for registered report protocols as studies for inclusion have already been selected.

Our Response: We appreciate the clarification regarding the requirements for Registered Report Protocols. As recommended by editor we have revised our manuscript to fit the criteria of a Study Protocol.

Specifically, we have clarified that data extraction has not yet been completed.

Revisions Undertaken

**Manuscript Type**

We have revised the manuscript to categorize it as a Study Protocol instead of a Registered Report Protocol.

**Clarification on Data Extraction:**

We have explicitly stated in the manuscript that data extraction has not yet been completed. This is now clearly mentioned in the Methods section.

**Confirmation**

We confirm that data extraction is not yet complete, and the revised manuscript accurately reflects the protocol for a systematic review study. We hope these revisions address the concerns raised, and we kindly request that our manuscript be considered for publication as a Study Protocol.

Regards,

Dr. Chaitali S. Vikhe
