# Supplementary material for: Effect of physiotherapy interventions on pain management, function and quality of life in patellofemoral pain syndrome: A systematic review protocol
Source: PLoS One. 2024 Dec 19;19(12):e0312279. doi: 10.1371/journal.pone.0312279 (PMC11658526; doi:10.1371/journal.pone.0312279)
Supplement: Attachment — Submitted filename: Respone to Reviwer.docx [file pone.0312279.s004.docx]

**Rebuttal Letter**

**To:** Academic Editor
PLOS ONE

**Date: 28/08/2024**

Thank you for the opportunity to revise and resubmit our manuscript titled "Effect of Physiotherapy Interventions on Pain Management, Function, and Quality of Life in Patellofemoral Pain Syndrome: A Systematic Review Protocol" (Manuscript ID: PONE-D-24-27461). We appreciate the valuable feedback provided by the reviewers and have carefully addressed each of their comments. Below, we provide a detailed point-by-point response to the reviewers' concerns, outlining the revisions made to the manuscript.

**Reviewer #1:**

1. **Title:**
   - **Comment:** The reviewer suggested changing "functional recovery" to "function" in the title.
   - **Response:** We have revised the title accordingly to "Effect of Physiotherapy Interventions on Pain Management, Function, and Quality of Life in Patellofemoral Pain Syndrome: A Systematic Review Protocol."
2. **Abstract:**
   - **Comment:** The reviewer pointed out an incomplete sentence: "Practice and future research, ultimately improving outcomes for individuals with PFPS."
   - **Response:** The sentence has been revised for clarity: "The findings of this review will guide clinical practice and future research, ultimately improving outcomes for individuals with PFPS."
3. **Introduction:**
   - **Comment:** The reviewer asked whether a meta-analysis or systematic review had been conducted in this field and how our study offers an advantage.
   - **Response:** Previous systematic reviews and meta-analyses have explored various treatment options for Patellofemoral Pain Syndrome (PFPS). However, no systematic review specifically addresses the combined effects of physiotherapy interventions on pain management, functional recovery, and quality of life in PFPS. Our study uniquely contributes to the field by focusing on the effectiveness of physiotherapy interventions tailored for runners, aggregating and analyzing diverse physiotherapy approaches within a single review framework. This approach enhances evidence-based decision-making in PFPS management and provides a clearer understanding of the comparative impacts of these interventions.
4. **Method:**
   - **Comment 1:** The reviewer requested specifying the type of interventions, study design, control group, variables studied, language, year of publication, and gray literature in the inclusion criteria.
   - **Response:** We have updated the Methods section to clearly specify the types of interventions (physiotherapy-based), study designs (randomized controlled trials), control groups (placebo, no intervention, or alternative interventions), variables studied (pain, function, quality of life), language (English), year of publication (2014–2024), and inclusion of gray literature.
   - **Comment 2:** The reviewer recommended including keywords in the search strategy and adding the Pedro database.
   - **Response:** We have revised the search strategy to include specific keywords and added the Pedro database to our list of sources.
   - **Comment 3:** The reviewer suggested focusing exclusively on randomized clinical trials (RCTs) and questioned the inclusion of all interventions without restriction.
   - **Response:** We have refined the inclusion criteria to focus exclusively on RCTs and physiotherapy interventions with a similar nature. This approach will reduce heterogeneity and facilitate a potential meta-analysis, ensuring that our research question is addressed effectively.
   - **Comment 4:** The reviewer noted the presence of grammatical errors.
   - **Response:** The manuscript has been thoroughly reviewed for grammatical errors, and necessary corrections have been made to ensure clarity and precision.

**Reviewer #2:**

1. **Abstract:**
   - **Comment:** Simplify complex sentences and clearly outline the objectives, methods, and anticipated outcomes.
   - **Response:** The language in the abstract has been simplified, and the objectives, methods, and anticipated outcomes are now clearly stated.
2. **Keywords:**
   - **Comment:** Write keywords based on MeSH terms.
   - **Response:** The keywords have been revised to align with MeSH terms, including "Patellofemoral Pain Syndrome," "Pain," "Function," "Quality of Life," and "Physiotherapy."
3. **Introduction:**
   - **Comment:** Strengthen the rationale by connecting the research question more directly to the existing literature and ensure each claim is backed by a current and relevant reference.
   - **Response:** The Introduction has been enhanced by directly linking our research question to the current literature, supported by up-to-date and relevant references.
4. **Methodology:**
   - **Comment 1:** Correct the use of "Google Scholar" as it is a search engine, not a database.
   - **Response:** "Google Scholar" has been replaced with appropriate databases such as Pedro, PubMed, and Cochrane Library.
   - **Comment 2:** Specify the time frame for study inclusion more clearly.
   - **Response:** The time frame for study inclusion has been specified as articles published between May 2014 to April 2024.
5. **Flowchart for Study Selection Process:**
   - **Comment:** Consider including a flowchart to depict the study selection process, which is common in systematic reviews.
   - **Response:** PRISMA-P flowchart has been included to depict the study selection process, ensuring transparency and replicability.
6. **Data Extraction Form:**
   - **Comment:** Provide a template or description of the form to be used for data extraction, explaining how it will be pilot-tested before full data extraction.
   - **Response:** We have included a description of the data extraction form and specified that it will be pilot-tested on a sample of studies before full data extraction to ensure consistency and accuracy.
7. **Risk of Bias Tool:**
   - **Comment:** Specify how you will handle studies with different designs (e.g., observational studies) that may not fit neatly into the Cochrane Risk of Bias tool. Also, consider discussing how you will address publication bias.
   - **Response:** We have clarified that our review will focus exclusively on RCTs, utilizing the Cochrane Risk of Bias tool. We will address publication bias using funnel plots and other statistical methods where appropriate.
8. **Discussion:**
   - **Comment:** Add a section on the expected contribution of your systematic review to the field and its potential to guide future research or clinical practice. Discuss possible challenges you anticipate during the review process, such as the heterogeneity of included studies.
   - **Response:** A section has been added to the Discussion outlining the expected contribution of our systematic review to the field, emphasizing its potential to guide future research and clinical practice. We have also discussed anticipated challenges, such as study heterogeneity, and how these will be addressed.
9. **References:**
   - **Comment:** Correct all references according to journal instructions.
   - **Response:** All references have been reviewed and corrected according to the journal's formatting requirements.

We believe that these revisions have significantly improved the manuscript, addressing all concerns raised by the reviewers. We are confident that the revised manuscript now meets the standards required for publication in PLOS ONE.

Thank you once again for your constructive feedback and for considering our revised submission. We look forward to your positive response.

Sincerely,
Chaitali S. Vikhe
Junior Resident, Department of Sports Physiotherapy
Ravi Nair Physiotherapy College
Datta Meghe Institute of Higher Education and Research (DU)
Sawangi (Meghe), Wardha-442004
Email: chaitalivikhe619@gmail.com
ORCID ID: 0009-0006-0677-6223
